# No-Touch Saphenous Vein - Vascular Damage and the London Connection

**DOI:** 10.21470/1678-9741-2022-0024

**Published:** 2022

**Authors:** Michael R. Dashwood

**Affiliations:** 1 Surgical and Interventional Sciences, Royal Free Hospital Campus, University College Medical School, London, United Kingdom. Department of Cardiac Surgery, Bristol Heart Institute, Bristol University, Bristol, United Kingdom

**Keywords:** Saphenous Vein, Vascular Damage, Coronary Artery Bypass, Microvessels, Muscle, Smooth, Vascular, Surgical Instruments, Review.

## Abstract

In this review, I summarise the circumstances leading to the collaboration between London and Örebro on the basic research performed to study potential mechanisms underlying the improved patency of saphenous veins harvested by the no-touch technique. Histological studies reveal various forms of vascular damage to saphenous vein grafts harvested in conventional coronary artery bypass grafting (CABG) whereas no-touch grafts retain a normal architecture. The perivascular fat that remains intact on no-touch saphenous vein grafts seems to play a particularly important role as the “protector” of all layers of the graft. In addition, the perivascular fat is a source of adipose cell-derived factors that may contribute to the success of the no-touch technique. While a number of trials have compared no-touch with conventional grafts following CABG, these have generally been limited to short follow-up periods, low patient numbers, and inadequate histological data. When handling no-touch saphenous vein at harvesting, there is no direct contact of the vein by surgical instruments, spasm does not occur, and high-pressure intraluminal distension is not required. While damage to both endothelial and vascular smooth muscle cells are evident at the microscopic and ultrastructural level in conventional saphenous vein grafts, their structure in no-touch grafts is preserved. Also, in no-touch veins, the vasa vasorum remains intact and transmural blood supply is maintained. This microvascular network is disrupted during conventional harvesting, a situation likely to stimulate processes involved in graft occlusion. The use of excess graft material for histology is to be encouraged for the assessment of vascular damage and even surgeon competence. If you don’t look, you don’t find.

**Table:** 

Abbreviations, Acronyms & Symbols	
ADRFs	= Adipocyte-derived relaxing factors
ADV	= Adventitia
CABG	= Coronary artery bypass grafting
CT	= Conventional
L	= Lumen
N	= Nucleus
NT	= No-touch
PVAT	= Perivascular adipose tissue
PVF	= Perivascular fat
sm	= Smooth muscle
SV	= Saphenous vein
TM	= Tunica media
VSMC	= Vascular smooth muscle vessel cells
VV	= Vasa vasorum

## INTRODUCTION

### A Meeting in Los Angeles: Keeping in Touch

Two years after the first publication describing the no-touch technique of saphenous vein harvesting^[^1^]^, I met Domingos Souza in Los Angeles, where we both presented posters at “The International Symposium on Vascular Protection: From Basic Science to the Clinic”. We exchanged e-mail addresses and kept in contact, and a year later, in October 1999, Domingos travelled from Orebro to London with his colleague, Derek Filbey, bringing with them a set of frozen saphenous vein samples from coronary artery bypass grafting (CABG) operations performed by their group. These samples were stored at -70°C over the weekend while I introduced Domingos and Derek to Hampstead, a popular part of north London where the Royal Free Hospital is situated. On the following Monday, while Domingos and Derek were on their flight back to Sweden, frozen sections of no-touch and conventional saphenous vein samples were being cut in the Dashwood lab and used for the identification of nitric oxide synthase employing the simple nicotinamide adenine dinucleotide phosphate-diaphorase histochemical technique.

**Fig. 1:**
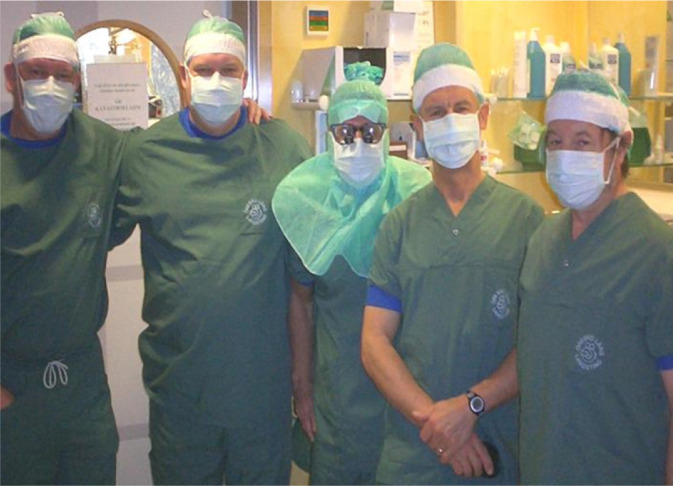
Preparing for a no-touch coronary artery bypass grafting operation in Örebro. A photograph taken on a trip to Örebro. Left to right: Mats Dreifaldt, David Abraham, Domingos Souza, Mick Dashwood, and Andrzej Loesch.

Early studies focused on the potential role of nitric oxide in the improved performance of no-touch saphenous vein grafts^[^2^,^3^]^. Here, apart from using histology, samples “imported” from Örebro were subjected to a variety of other techniques including immunohistochemistry, western blot analysis, reverse transcription-polymerase chain reaction, and the biochemical assessment of nitric oxide synthase activity. Excellent, parallel, histochemical studies at this stage were also performed at the Pathology Department in Örebro University Hospital. These studies led to a number of publications in peer reviewed journals^[^2^-^6^]^ as well as presentations at many international scientific meetings. Our early publications and the increasing interest in the no-touch technique played an important part in obtaining a British Heart Foundation Project Grant to Janice Tsui and myself. This funding provided equipment and consumables and the ability to visit Örebro frequently (Figure 1). Furthermore, data generated from these studies contributed to the basic research sections in the PhD theses of Domingos Souza and Mats Dreifaldt, who both spent time in my laboratory in London.

## DISCUSSION

### Vascular Damage

**Fig. 2:**
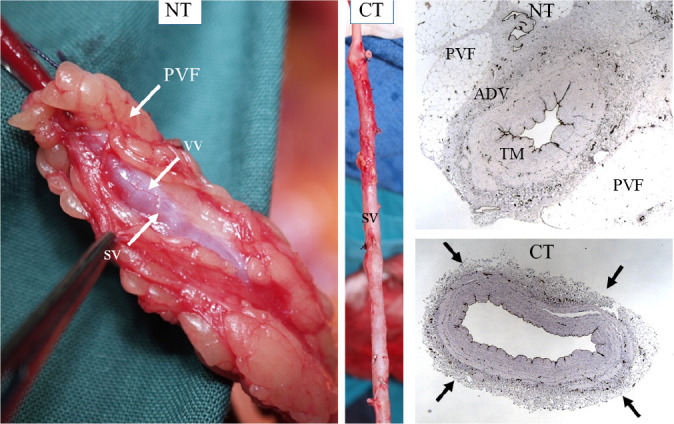
Vascular damage; no-touch (NT) versus conventional (CT) saphenous vein (SV). Left panel shows explants of NT and CT SV used for coronary artery bypass grafting. The perivascular fat (PVF) surrounding the SV remains intact, and the superficial adventitial vasa vasorum (VV) is visible in the NT SV. The CT SV has PVF removed, and adventitia (ADV) is stripped off or partially damaged. Right panel shows transverse sections of NT (top) and CT (lower) SV stained with haematoxylin and eosin and endothelial cells identified using CD31. The PVF and ADV remain intact on NT SV, but PVF is removed on CT SV. The vessels wall is thicker in NT than CT SV. A high proportion of the ADV and associated VV (dark punctate staining) is damaged/removed in CT SV (arrows). TM=tunica media.

After processing vessel sections, there were obvious differences between the no-touch *vs.* conventional saphenous veins. The most striking was the uninterrupted pattern of endothelial staining that lined the lumen of no-touch veins, but that was fragmented in the conventional vein samples. Traditional histology methods were used that revealed dramatic differences between no-touch and conventional veins: in no-touch saphenous veins, the surrounding cushion of perivascular fat remained intact, the lumen exhibited folding, and the endothelial lining was intact (Figure 2). By comparison, conventional samples exhibited signs of damage with the perivascular fat removed, a dilated lumen showing regions of endothelial denudation, and a thin media when compared with no-touch veins (Figure 2). Thus began the Anglo/Swedish collaboration between colleagues at the Royal Free Hospital/University College London and our friends at Örebro University Hospital, a collaboration lasting over 20 years.

**Fig. 3:**
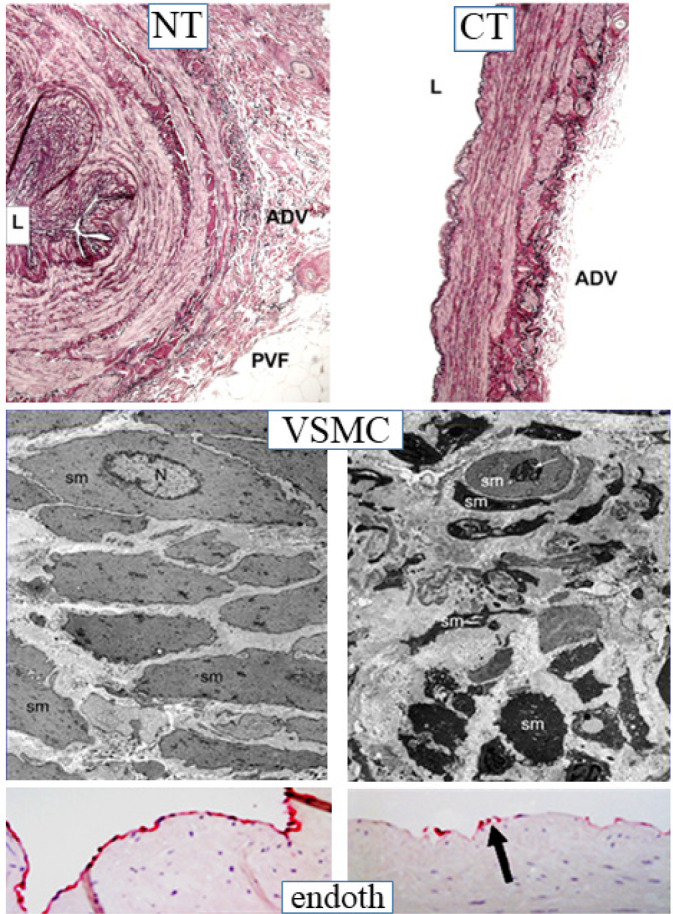
No-touch (NT) versus conventional (CT) saphenous vein (SV); vascular smooth muscle and endothelial cells. Top left panel: Part of a transverse section of NT SV where the lumen (L) is folded, the adventitia (ADV) and perivascular fat (PVF) remain intact, and the vessel wall is thick. Top right panel: In the CT SV, the PVF is removed, and the ADV is partially removed and/or damaged. Middle left panel: A representative example of a transmission electron micrograph showing the ultrastructure of vascular smooth muscle vessel cells (VSMC) within the media of NT SV that are of regular size and uniform shape. Middle right panel: VSMCs in the media of CT SV exhibit polymorphism and are ovoid, elongated, or multishaped. (Both from Ahmed et al.^[^7^]^, 2004). Lower left panel: The luminal endothelium (red staining) of NT SV is continuous. Lower right panel: The endothelium in CT SV is damaged with only few cells remaining intact (arrow). N=nucleus; sm=smooth muscle.

**Fig. 4:**
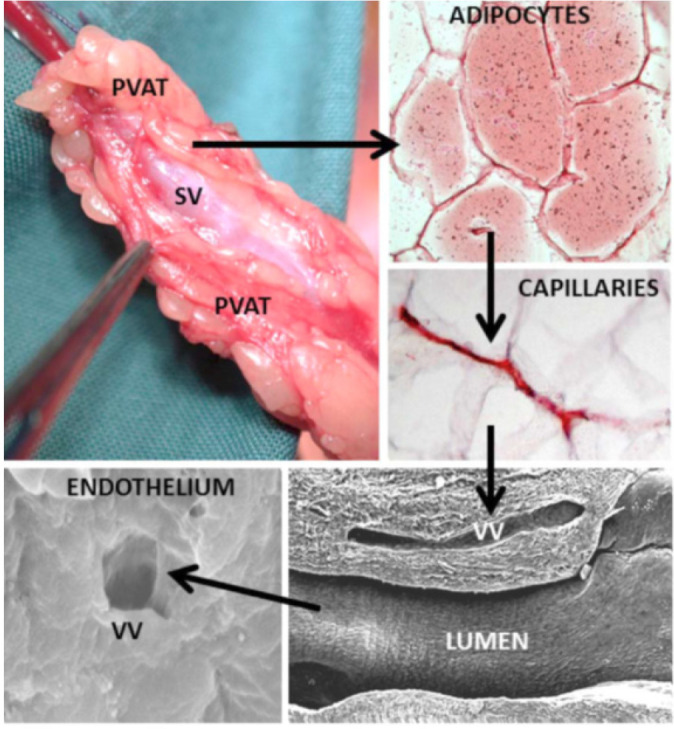
Potential transport of perivascular fat-derived factors in no-touch (NT) saphenous vein (SV). Top left: A length of NT SV harvested for coronary artery bypass grafting with perivascular adipose tissue (PVAT) intact. Top right: PVAT adipocytes exhibiting positive immunostaining (red) for endothelial nitric oxide synthase. Below is the capillary network within PVAT. Lower right: Longitudinal scanning electron microscopic image of vasa vasorum (VV) terminating close to the vein lumen. Lower left: Scanning electron microscope image of an aperture/termination of VV at the luminal endothelium. Arrows indicate possible transport from PVAT via the media to the vessel lumen, which may be bi-directional. (From Fernandez-Alfonso et al.^[^13^]^).

While preliminary studies provided evidence for a role of nitric oxide in the improved patency of no-touch saphenous vein grafts, an additional observation was the striking difference in morphology when comparing no-touch saphenous veins with those prepared by the conventional method (Figures 2 and 3)^[^4^,^5^,^7^]^. Essentially, no-touch-harvested saphenous veins retain a normal architecture where the perivascular cushion of fat is preserved and, since no spasm occurs, high-pressure intraluminal distension is not required, and the endothelium remains intact. By comparison, conventional grafts exhibit various aspects of vascular damage due to a combination of surgical trauma and high-pressure intraluminal distension (Figure 3). While these differences are obvious, both on visual examination and at the microscopic level, the variations are dramatic when observed at the ultrastructural level. Here, employing both scanning and transmission electron microscopy, Andrzej Loesch et al. described striking changes to endothelial and vascular smooth muscle cells of conventional compared with no-touch saphenous veins (Figure 3)^[^6^,^8^,^9^]^. Of particular interest, and of potential importance regarding vein graft performance, were novel findings concerning the vasa vasorum. At the light microscope level, many sections of no-touch saphenous veins exhibit elongated folds of the lumen taken to represent potential “channels” that may signify termination of the vasa vasorum. Using scanning electron microscopy, small apertures were observed within regions of the luminal endothelium, adding further support to this suggestion (Figure 4)^[^9^,^10^]^. These observations raise the possibility of a novel microvascular network that extends throughout the vein wall and that connects the innermost with the outermost vessel layers. Such a system would explain the retrograde filling of blood in the adventitial vasa vasorum observed when removing vascular clamps at completion of no-touch saphenous vein graft implantation, as described by Domingos Souza^[^11^]^. Interestingly, using transmission electron microscopy, collapse of vasa vasorum in the media and occlusion by clumps of erythrocytes were observed in conventionally prepared saphenous veins, effects presumably due to a combination of surgical trauma and high-pressure distension. By comparison, the vasa vasorum in no-touch saphenous veins appeared patent and contained individual erythrocytes of normal appearance^[^8^]^. Taken together these observations suggest that in no-touch saphenous vein grafts medial blood flow is preserved and that, at least during early stages after graft insertion, the oxygen and nutrient requirements of the graft wall are maintained. This is in contrast to conventional grafts where damage to the vasa vasorum reduces or prevents transmural blood flow causing medial ischaemia, a condition leading to neointimal hyperplasia, atheroma formation, and eventual graft failure^[^10^]^.

### Perivascular Fat: FETTVEN

**Fig. 5:**
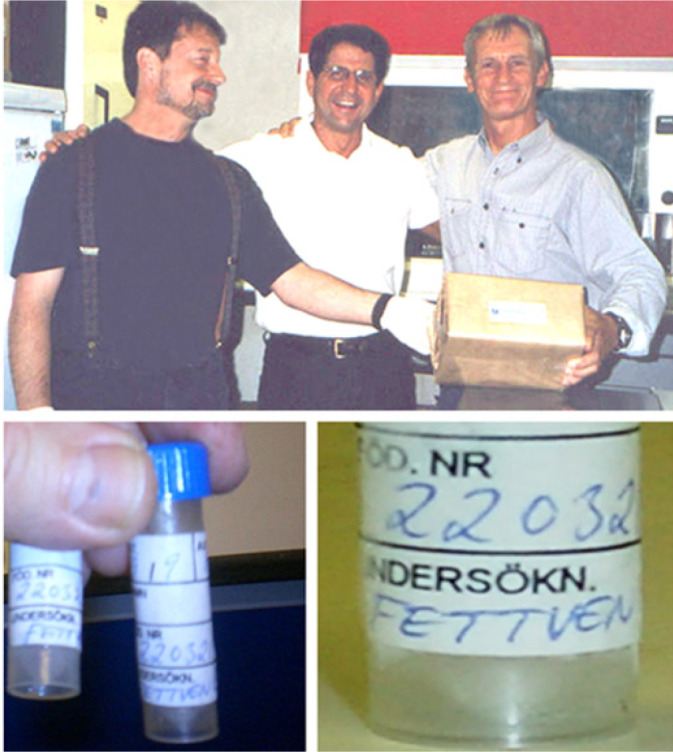
A delivery of ‘FETTVEN’. Top: Domingos Souza (middle) delivers samples of saphenous vein collected in Örebro. Andrzej Loesch (left) and Mick Dashwood (right) accept this gift. Lower left: Tubes containing saphenous vein samples. Lower right: No-touch saphenous vein tube labelled ‘FETTVEN’.

Another important factor contributing to the success of no-touch saphenous vein grafts is the preservation of perivascular fat that remains intact at harvesting but is removed when using the conventional method. Initially, Souza realised that by handling the saphenous vein by this cushion of fat, direct contact with the vein by surgical instruments was avoided, spasm prevented, and high-pressure manual distension was not necessary. As a result, damage to all vein layers was reduced, thus the vein retained its normal architecture. Also, this cushion of perivascular fat provides mechanical support for the graft, a situation of particular importance in preventing kinking in grafts of excessive length^[^11^]^. For over two decades, there has been increased interest in the protective action of perivascular fat-derived factors, in particular those possessing vasodilator properties (via adipocyte-derived relaxing factors [ADRFs]). Using a combination of histological and biochemical techniques, it was shown that the perivascular fat surrounding no-touch saphenous veins exhibits nitric oxide synthase immunostaining, nitric oxide synthase protein, and the ability to generate nitric oxide. Accordingly, in addition to the presence of adiponectin and leptin (both ADRFs), it is proposed that by preserving the perivascular fat of the saphenous vein, “beneficial” perivascular fat-derived factors may play an additional role in the improved performance of no-touch grafts, either by a direct action on the adjacent adventitia or via the vasa vasorum^[^12^]^ (Figure 4). Much of the work relating to the role of perivascular fat has been performed either in collaboration or with expert advice from Professor Marisol Fernandez-Alfonso, in Madrid^[^13^]^. Looking back and relating to the “nomenclature” of the no-touch saphenous vein, it is noteworthy that early samples provided by colleagues in Örebro were aptly labelled ‘FETTVEN’, Swedish for fat vein (Figure 5).

### Other No-Touch Trials and Studies

To date, this Anglo/Swedish collaboration has generated most of the published information comparing the morphology of no-touch and conventional saphenous vein preparations used for CABG and the effects of surgical trauma. The vascular damage inflicted to the vein is readily revealed by routine histology methods. Various strategies aimed at improving the patency of the saphenous vein as a bypass conduit have been previously documented, although these have been mainly performed using animal models^[^14^-^16^]^. The few published trials evaluating no-touch *vs.* conventional saphenous vein grafts have been of limited value and lack the necessary histological examples of harvested material^[^16^,^17^]^. As a result, one is unable to reliably judge any differences between preparations or whether veins have indeed been harvested using Souza’s no-touch technique correctly^[^1^,^12^]^. When performing CABG, it may be advisable for samples of surplus graft to be routinely sent to the local pathology laboratory in order to assess vessel morphology, evidence of vascular damage, and even surgeon competence.

When comparing conventional with no-touch vein grafts in CABG patients, there are striking differences, particularly regarding removal of the perivascular fat, damage to the endothelium, disruption of the vasa vasorum, and ultrastructural shape changes of both endothelial and vascular smooth muscle cells. Clearly, many surgeons appear oblivious to this damage and, as a result, “a damaged vein is being used to repair a damaged heart”. Since its introduction, an increasing number of cardiac centres worldwide have adopted the no-touch method of saphenous vein harvesting. Only few trials have directly compared no-touch and conventional saphenous vein patency, although these have been on low patient numbers and of poor design^[^17^,^18^]^. However, a recent multi-centre study in China provides strong support for the no-touch technique. Here, 1,337 patients receiving no-touch saphenous vein grafts were compared with 1,318 patients receiving conventional grafts, where occlusion rate was significantly lower in no-touch *vs.* conventional grafts. Furthermore, recurrence of angina was significantly lower in the no-touch than in the conventional group^[^19^]^. Apart from data published by the Örebro group, trials have been limited to short follow-up times.

### The Brazilian Link

**Fig. 6:**
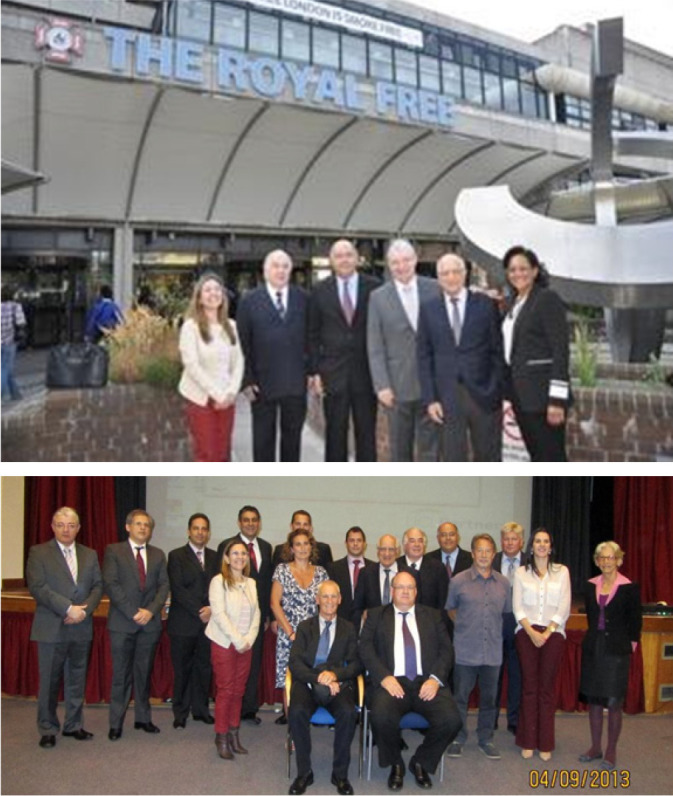
The 2^nd^ Anglo/Brazilian meeting. Royal Free Hospital, Hampstead, London. Top panel (left to right): Professors Tania Maria de Andrade de Rodriques, Otoni Moreira Gomes, João Batista Vieira de Carvalho, Melchior Luis Lima, Elias Kallas, and Antoinette Oliveira Blackman at the main entrance of the Royal Free Hospital. Lower panel: Organisers and presenters at the 2^nd^ Anglo/Brazilian meeting, September 2013. (I am grateful to Melchior Lima for providing these photographs.)

Fortunately, the popularity of the no-touch technique in Brazil gave me the opportunity of attending and presenting updates of our studies at the Annual Scientific Forum of the International Congress of Cardiovascular Sciences held at various states in Brazil organized by Professor Otoni Gomes and, more recently, by Professor Melchior Lima. In return, and with the help of Professor David Abraham, three very memorable Anglo/Brazilian meetings were held at the Royal Free Hospital (Figure 6). This provided the opportunity, not only to exchange ideas, but also to introduce our Brazilian friends to an important part of English culture - enjoying beer at The Magdala Tavern, a popular local Pub. So, in addition to the Anglo/Swedish London connection, an Anglo/Swedish/Brazilian London connection has also developed over the years.

Research throughout the world has been seriously affected in the last two years by the coronavirus disease 2019 (or COVID-19) situation, particularly that involving patients undergoing many different forms of surgery, including CABG. Travel restrictions imposed due to the virus have also had an impact on scientific meetings that have been mainly restricted to online conferences and webinars. We all look forward to eventually returning to normal, reuniting with old friends and colleagues and making new acquaintances. My own experience is that chance encounters can lead to long-term friendship and productive collaboration such as that I have enjoyed since my involvement with Domingos and fellow “no-touchers”. Also, in this era of multi-authored publications, I remember a maxim of my old boss, Professor Wilhelm Feldberg, who believed that “you should be able to recognise your fellow co-authors when passing them in the corridor”. While that was mainly true in the 1970s, I doubt it is today.

## CONCLUSION

For the no-touch technique and for the benefit of patients undergoing CABG, it is important that follow-up of ongoing and future studies is extended over longer periods. I would also encourage those participating in such trials to take advantage of access to surplus saphenous vein segments and to use these samples for further basic research. There is much to be gained using this otherwise wasted material. There is a danger that in an era when advances in bypass surgery, ranging from robotics to the use of synthetic materials and gene targeting, the art of observation is lost. Despite the various contemporary efforts into improving myocardial revascularization procedures, it is important to consider the quotation of Albert Einstein, “imagination is more important than knowledge” - a line that appears at the beginning of Domingos Souza’s PhD thesis^[^20^]^.

**Table:** 

Authors’ Roles & Responsibilities	
MRD	Substantial contributions to the conception or design of the work; or the acquisition, analysis, or interpretation of data for the work; drafting the work or revising it critically for important intellectual content; agreement to be accountable for all aspects of the work in ensuring that questions related to the accuracy or integrity of any part of the work are appropriately investigated and resolved; final approval of the version to be published
